# Nitrifying biofilms deprived of organic carbon show higher functional resilience to increases in carbon supply

**DOI:** 10.1038/s41598-020-64027-y

**Published:** 2020-04-28

**Authors:** Sharada Navada, Maja F. Knutsen, Ingrid Bakke, Olav Vadstein

**Affiliations:** 1 Department of Chemistry, NTNU - Norwegian University of Science and Technology, N-7491 Trondheim, Norway; 2 Krüger Kaldnes AS (Veolia Water Technologies), N-3241 Sandefjord, Norway; 3 Department of Biotechnology and Food Science, NTNU - Norwegian University of Science and Technology, N-7491 Trondheim, Norway; 4 Present Address: Oxy Solutions, Gaustadalleen 21, N-0349 Oslo, Norway

**Keywords:** Applied microbiology, Microbial ecology, Biofilms

## Abstract

In nitrifying biofilms, the organic carbon to ammonia nitrogen (C/N) supply ratio can influence resource competition between heterotrophic and nitrifying bacteria for oxygen and space. We investigated the impact of acute and chronic changes in carbon supply on inter-guild competition in two moving bed biofilm reactors (MBBR), operated with (R1) and without (R0) external organic carbon supply. The microbial and nitrifying community composition of the reactors differed significantly. Interestingly, acute increases in the dissolved organic carbon inhibited nitrification in R1 ten times more than in R0. A sustained increase in the carbon supply decreased nitrification efficiency and increased denitrification activity to a greater extent in R1, and also increased the proportion of potential denitrifiers in both bioreactors. The findings suggest that autotrophic biofilms subjected to increases in carbon supply show higher nitrification and lower denitrification activity than carbon-fed biofilms. This has significant implications for the design of nitrifying bioreactors. Specifically, efficient removal of organic matter before the nitrification unit can improve the robustness of the bioreactor to varying influent quality. Thus, maintaining a low C/N ratio is important in nitrifying biofilters when acute carbon stress is expected or when anoxic activity (e.g. denitrification or H_2_S production) is undesirable, such as in recirculating aquaculture systems (RAS).

## Introduction

The nitrification process is widely used in wastewater treatment and recirculating aquaculture systems (RAS) for the aerobic conversion of ammonia to nitrate. This process is performed by autotrophic bacteria that oxidize ammonia to nitrite (ammonia oxidizing bacteria, AOB) and subsequently, nitrite to nitrate (nitrite oxidizing bacteria, NOB). Microorganisms capable of complete ammonia oxidation to nitrate also exist within the genus *Nitrospira*^1^. Nitrification is commonly achieved in biofilm systems, as they enable the higher biomass retention times necessary for the slow-growing nitrifiers. The nutrient and oxygen gradients in biofilms create niches that allow several microbial guilds to coexist^2^. Therefore, life in a biofilm not only facilitates mutualism (e.g., between the AOB and NOB), but can also induce competition for resources, especially oxygen.

In biofilms, nitrifying bacteria coexist with heterotrophic bacteria^3^. Heterotrophs can be beneficial to the nitrifiers by maintaining the biofilm structure through EPS secretion^4,5^. This protects the nitrifying bacteria against detachment and dehydration^6^. In turn, the soluble microbial products produced by the nitrifiers and dead cells can support the growth of the heterotrophs, especially in the absence of other organic carbon sources^3^. However, if the oxygen diffusion rate into the biofilm is lower than the consumption rate by the bacteria, competition for oxygen will ensue. Owing to their higher growth rates and lower threshold concentration of oxygen for growth, heterotrophs are superior competitors and can suppress nitrification activity^3,7–9^. In a given system, the inter-guild interaction between heterotrophs and nitrifiers may be dominantly mutualistic or competitive depending on several factors. These factors include oxygen and space availability^9–11^, specific loading rate of degradable carbon^12,13^, biofilm structure^14^, and organic carbon: ammonia nitrogen (C/N) ratio^7,15^.

In a biofilm, increased carbon supply can lead to higher competition for oxygen and thus inhibit nitrification^9^. Moreover, an increase in the C/N ratio can not only reduce the nitrification efficiency but also increase denitrification^16^. While this may be advantageous in many systems, it may also be undesirable in certain situations. In WWTP, denitrification in the nitrifying unit can lead to insufficient nitrate production, which can render the pre-denitrification stage anaerobic, leading to a negative feed-back loop that can eventually stop nitrification^17^. In biofilm systems treating seawater effluents, increased C/N can lead to sulphate reduction, producing H_2_S, a hazardous gas. In RAS, sulphate reduction can be extremely detrimental as H_2_S is toxic to fish at concentrations as low as a few ppb^18^. Therefore, it is necessary to better understand the response of nitrifying bioreactors to increases in the C/N ratio.

The response of nitrifying bioreactors to acute and chronic increases in carbon supply is poorly understood, especially in terms of the biofilm functionality and the microbial community composition. Most studies have investigated either short-term or long-term effects of the C/N ratio, but not both^4,7,13,19,20^. Therefore, we compared the impact of acute and chronic increases in organic carbon supply on two nitrifying moving bed biofilm reactors (MBBR). The MBBRs had been operated at C/N mole ratios of 0 (autotrophic) and 1 (carbon-fed), respectively. We predicted that reactors not fed with organic carbon would better handle increases in carbon supply due to higher nitrifying to heterotrophic biomass ratios.

## Materials and Methods

### Experimental design and setup

The experiment consisted of two continuously operated nitrifying MBBRs with three operating phases over 72 days (Table 1). In phase 1, reactors R0 and R1 were operated at influent C/N mole ratios of 0 and 1, respectively. In this phase, acute carbon inhibition tests were performed (Section 2.2). Phase 2 was designed to investigate the effect of an increase in the C/N ratio. Thus, on day 52, the C/N ratio in both reactors was increased to 3 by refilling both reactors with a new synthetic medium (C/N = 3) and resuming continuous operation with this new medium. In phase 3, the medium flow rates to both reactors were doubled (on day 59), while maintaining the same influent C/N of 3. In this phase, we investigated the combined impact of a chronic increase in the C/N and the loading rate of ammonia and organic carbon. In phases 2 and 3, both reactors had similar operating conditions. Unless otherwise mentioned, the term ammonia denotes total ammonia nitrogen (NH_3_-N + NH_4_^+^-N).

**Table 1:** Experimental design: Influent C/N mole ratio in MBBRs R0 and R1 during the experimental phases, with the corresponding specific ammonia loading rates and hydraulic retention times (HRT).

Phase	Influent C/N mole ratio		Ammonia loading rate (gN m^−2^ d^−1^)	HRT (hours)	Days
	R0 (autotrophic)	R1 (carbon-fed)			
Phase 1	0	1	0.78	12.9	1–2
Phase 2	3	3	0.78	12.9	52–59
Phase 3	3	3	1.53	6.3	59–72

The experimental setup contained two 0.6 L aerated glass reactors filled (50% by volume) with biofilm carriers (AnoxK^TM^1, AnoxKaldnes, Sweden)^21^. The carriers were started on a synthetic medium without organic carbon. Thereafter, reactors R0 and R1 were continuously operated on influent C/N of 0 and 1, respectively, for four months before the experiment. The experiment was performed at 25 °C, pH 7.5. The medium had an ammonia concentration of 101 ± 3 mgN L^−1^. Each litre of medium had 0.47 g (NH_4_)_2_SO_4_, 1.00 g NaHCO_3_, and 0.40 g K_2_HPO_4_, with 10 mL micronutrient solution containing (in g L^−1^) 2.5 MgSO_4_·7H_2_O, 1.5 CaCl_2_·2H_2_O, 0.2 FeCl_2_·4H_2_O, 0.55 MnCl_2_·4H_2_O, 0.068 ZnCl_2_, 0.12 CoCl_2_·6H_2_O, 0.12 NiCl_2_·6H_2_O, and 2.8 EDTA. The C/N mole ratios were set to 1 and 3 as required, by adjusting the sodium acetate concentration in the medium to 3.6 and 11 mM, respectively (corresponding C/N mass ratios of 0.9 and 2.6). Unless otherwise specified, the C/N ratios refer to mole ratios.

### Maximum ammonia oxidation rate (AOR_max_)

On days 10 and 11, batch tests were conducted to compare the maximum ammonia oxidation rate (AOR_max_). Biofilm carriers (100 mL) were transferred from each MBBR to another similar MBBR containing 500 mL of medium. In the first test, the medium in both batch MBBRs had a C/N ratio of 0, whereas in the second test, it had a C/N ratio of 1. During each test, ammonia concentration was measured every 60 minutes for 5–7 hours.

### Inhibition of nitrification by organic carbon

On day 20, tests were conducted to assess the dose-dependent response of the nitrifiers to acute carbon stress. For this, 100 mL of carriers from R0 and R1, respectively, were transferred to two similar batch MBBRs. The nitrification performance was measured at different acetate concentrations: 0, 10, 31.6, 100, 316, and 1000 mM. The test was started by filling each reactor with 500 mL medium containing 50 mg NH_4_^+^-N L^−1^ and no acetate. The nitrification capacity was determined as the maximum nitrate production rate (NPR_max_) by measuring the nitrate concentration every 30 minutes for three hours. Ammonium and nitrite concentration were measured at the beginning and at the end of the test. The same procedure was repeated by refilling the MBBRs each time with a new medium with a higher acetate concentration.

### Physicochemical analyses

Ammonia, nitrite, and nitrate concentrations in the MBBRs were analysed every 1–3 days (with few exceptions). On the first days of phases 2 and 3, ammonia, nitrite, nitrate, acetate, and oxygen were measured every 60 min for 8–9 hours to determine the short-term impact of the change. Acetate concentration was analysed every 1–4 days between days 45–72. For all analyses except oxygen, the water samples were filtered through 0.45 µm polyether sulfone membrane filters and analysed with the respective Hach Lange cuvette kits and a DR3900 VIS spectrophotometer (Hach Lange, Germany). Dissolved oxygen concentration was measured using a microfiber optic oxygen transmitter (Fibox 4, PreSens, Germany).

### Microbial analysis

#### Biofilm microbial community composition

One biofilm carrier from each reactor was sampled every 2–7 days after day 15 and stored at −20 °C until analyses. The microbial community composition in the biofilm was determined by 16 S rRNA gene amplicon sequencing. The biofilm DNA was extracted using PowerSoil^®^ DNA Isolation kit (Mo Bio Laboratories, USA). Amplicons encompassing the v3 and v4 region of the 16 S rRNA gene were generated using the primers Ill338F (5’-TCGTCGGCAGCGTCAGATGTGTATAAGAGACAGNNNNCCTACGGGWGGCAGCAG-3’) and Ill805R (5’- GTCTCGTGGGCTCGGAGATGTGTATAAGAGACAGNNNNGACTACNVGGGTATCTAAKCC-3’). The PCR reactions were run for 33 cycles (98 °C 15 s, 55 °C 20 s, 72 °C 20 s) with 0.3 µM of each primer, 0.25 mM of each dNTP, 2 mM MgCl_2_, Phusion Hot Start II High-Fidelity DNA Polymerase and Phusion buffer HF (Thermo Fisher Scientific, USA) in a total volume of 20 µL. To achieve equal concentrations of the PCR amplicons for each sample, they were normalized using the SequalPrep^TM^ Normalization Plate Kit (Invitrogen, Thermo Fisher Scientific, USA). A second PCR was performed to attach index sequences to the normalized amplicons using the Nextera XT Index Kit. The reactions were run for eight cycles (98 °C 15 s, 50 °C 20 s, 72 °C 20 s) with 2.5 µL of each index primer in a total volume of 25 µL. All the other reaction conditions were as described for the first PCR. The indexed PCR products were normalized as described above, pooled, and concentrated using Amicon^®^ Ultra-0.5 Centrifugal Filter Devices. The concentrated sample was sequenced on a MiSeq lane with v3 reagents employing 300 bp paired end reads at the Norwegian Sequencing Centre. The Illumina sequencing data are available at the European Nucleotide Archive (accession number: PRJEB37773).

### Data analysis

#### Physicochemical analysis

During continuous operation, the ammonia oxidation efficiency (AOX) was calculated based on the influent and effluent ammonia concentration. The nitrite oxidation efficiency (NOX) was calculated from the nitrate produced per mass of ammonia oxidized. During the batch tests, AOR_max_ or NPR_max_ were calculated from the slopes of the regression lines between the parameter (ammonia or nitrate) and time. These rates were compared using analysis of covariance (ANCOVA)^22,23^. Normality of the residuals was checked by Shapiro-Wilk tests. Dose-response curves were fitted between percent inhibition of NPR_max_ and acetate concentration using a two-parameter log-logistic model^24^. The ED_50_ (effective dose of acetate for 50% inhibition of NPR_max_) was thereby determined. Differences were considered statistically significant at a 95% confidence interval (*p* < 0.05). Data analysis was performed in R (V3.6.1).

#### Analysis of sequences

The Illumina sequencing data were processed using the USEARCH pipeline (version 10; https://www.drive5.com/usearch/). The command Fastq_mergepairs was used for merging paired reads, trimming off primer sequences, and filtering out reads shorter than 400 bp. The processing further included demultiplexing and quality trimming (Fastq_filter command with expected error threshold of 1). Chimera removal and clustering at 97% similarity level was performed using UPARSE-OTU algorithm^25^. Taxonomic assignment was performed by applying SINTAX script^26^ with a confidence threshold of 0.8 and the RDP reference data set (version 16). Archaea and operational taxonomic units (OTU) that were undefined at the domain level were removed from the data, as the primers were not designed for archaea. Chloroplasts/cyanobacteria were also removed. To evaluate the sequencing depth, we compared the estimated taxa richness (Chao1) to the observed number of OTUs. The OTU table was normalized to the sum of sample reads. Thereafter, OTUs with a maximum of less than 0.1% in any sample were removed. From the resulting OTU table, the α-diversity of each sample was calculated using the first-order diversity number, taxa richness, and evenness^27^. Ordination by principal coordinate analysis (PCoA) was performed to compare samples based on Bray-Curtis and Sørensen-Dice indices (β-diversity). Permutational multivariate analysis of variance (PERMANOVA) based on Bray-Curtis distances was used to test the hypothesis of equal community composition between groups of samples^28^. Similarity percentages (SIMPER) was used to determine the contribution of individual OTUs to the dissimilarity between samples^29^. Microbial data analysis was performed in R (V3.6.1) using packages phyloseq and vegan^30,31^.

To identify the OTUs representing potential nitrifying bacteria, we inspected the OTU table for OTUs that were classified in taxa known to include ammonia- and nitrite- oxidizing bacteria. We used the Ribosomal Database Project (RDP) tools Classifier^32^ and SeqMatch (restricted to “Type strains”) and SILVA ACT (Alignment, Classification and Tree service)^33^ to analyse these OTU sequences. Further, to examine the relationship between the OTUs classified as *Nitrosomonadaceae* in this study with previously described *Nitrosomonas* species, we performed a neighbour-joining phylogenetic analysis using the Multi-way alignment tool with default parameters in Clone Manager Professional version 9 (v9.51). We thereby compared the OTUs in our study with 16 S rRNA gene sequences from the RDP database for the following *Nitrosomonas-*type strains: *N. europaea* (S000384490*), N. eutropha* (S000396471), *N. ureae* (S002967454), *N. marina* (S002967455), *N. halophila* (S002967457), *N. aestuarii* (S002967458), *N. nitrosa* (S002967459), *N. oligotropha* (S002967460), and *N. communis* (S003288146).

## Results

### Nitrification in R0 was greater than in R1 after a chronic increase in carbon supply

**Figure 1:**
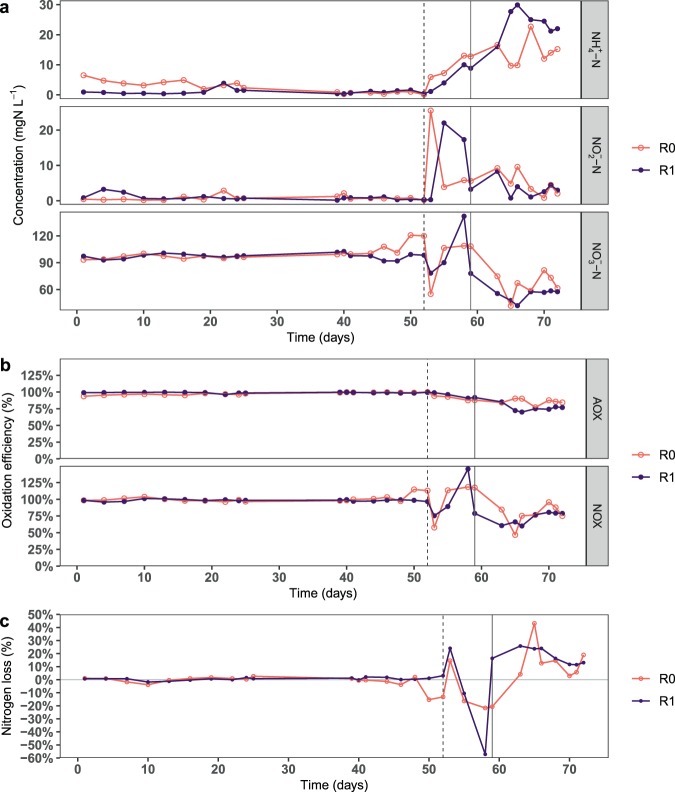
Nitrification performance in reactors R0 and R1 during the experimental period. (**a**) Ammonia, nitrite, and nitrate concentration, (**b**) ammonia and nitrite oxidation efficiency (AOX and NOX, respectively), and (**c**) nitrogen mass balance. During phase 1 (days 1–52), R0 and R1 were operated at influent C/N ratios of 0 and 1, respectively. Phase 2 was started on day 52 by increasing the influent C/N ratio to 3 in both reactors (dashed line). Phase 3 was started on day 59 by doubling the medium flow rate to both reactors (vertical solid grey line). The nitrogen loss was calculated as the difference between the influent ammonia concentration (~101 mgN L^−1^) and the total effluent nitrogen species concentration (NH_4_^+^-N, NO_2_^−^-N, and NO_3_^−^-N). In phase 2, the flow rates were irregular and larger volumes of water were extracted for analyses, resulting in large variations in the nitrogen mass balance.

During phase 1 (days 1–52), despite the difference in carbon supply, both reactors had similar nitrification performance with AOX and NOX of 98–100% (Fig. 1a,b). Further, the AOR_max_ of the treatments in media with C/N ratios of 0 and 1 was not significantly different (*p* = 0.27) (Supplementary Fig. S1).

**Figure 2:**
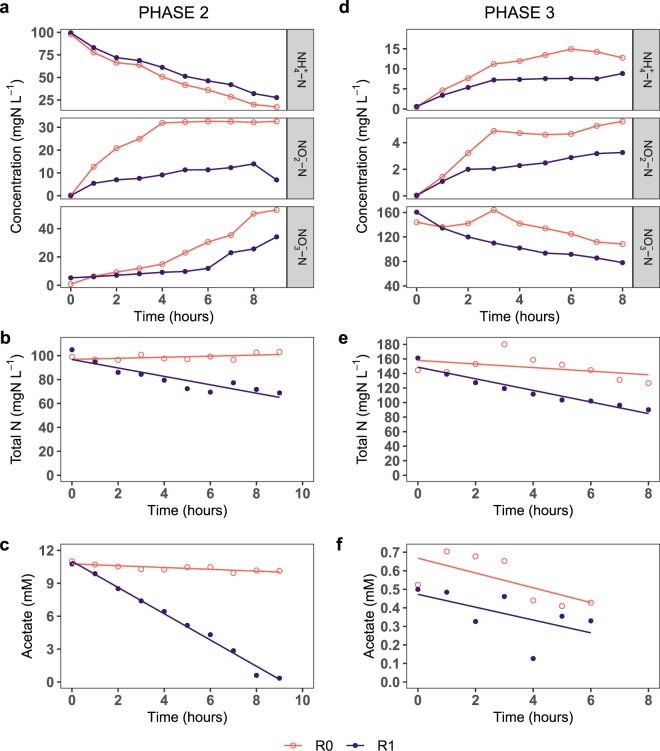
Graphs show the short-term response during the first days of phases 2 (a,b,c; C/N increased to 3) and 3 (d,e,f; medium flowrate doubled). Graphs show, as a function of time: (**a**,**d**) ammonia, nitrite, and nitrate concentration; (**b**,**e**) nitrogen mass balance as the sum of inorganic nitrogen species concentration (NH_4_^+^-N, NO_2_^−^-N, and NO_3_^−^-N); and (**c**,**f**) acetate concentration. Note the different y-axes scales.

In the first 9 h of phase 2 (day 52, C/N increased to 3), R0 had higher nitrification activity than R1. This is indicated by the lower ammonia concentration and higher nitrate concentration in R0 than in R1 (Fig. 2a). Overall, the sum of the inorganic nitrogen species decreased significantly in R1 (nitrogen loss of 3.4% h^−1^), whereas it was constant in R0 (Fig. 2b). The nitrogen loss was likely due to denitrification, as it was accompanied by a decrease in acetate concentration, which was also significantly higher in R1 (1.2 mM h^−1^) than in R0 (0.08 mM h^−1^) (Fig. 2c). The oxygen concentration decreased only slightly by 0.3–0.5 mg L^−1^ (Supplementary Fig. S2 B). Throughout phase 2, the ammonia concentration in R1 was lower than in R0, except on day 52 (Fig. 1a). During this phase, the flow rates were irregular and larger volumes of water were extracted for analyses, resulting in large variations in the nitrogen mass balance.

In the first 8 h of phase 3 (doubled medium flowrate), R1 had lower concentration of all N-species than R0, suggesting significant denitrification activity (Fig. 2d). Denitrification was observed in both reactors, with nitrogen losses in R0 and R1 being 1.7 and 4.9% h^−1^, respectively (Fig. 2e). The acetate concentration also decreased with time in both reactors, albeit with a weak linear correlation (adjusted R^2^ < 0.35) (Fig. 2f). The oxygen concentration decreased from 7.1 to ~6.5 mg L^−1^ (Supplementary Fig. S2 C).

In long-term operation during phase 3, the nitrification activity decreased in both reactors, and the ammonia and nitrite concentration increased (Fig. 1a). In this period, R0 had higher nitrification activity than R1. The average AOX in R0 and R1 was 86 and 78%, respectively, whereas the average NOX was 82 and 73%, respectively (Fig. 1b). Both reactors showed denitrification activity, as the NH_4_^+^-N conversion rate (sum of autotrophic and heterotrophic activity) was greater than the total production rate of NO_2_^−^-N and NO_3_^−^-N (average nitrogen loss 16 ± 11%, Fig. 1c). The oxygen concentration decreased linearly by 0.5–0.7 mg L^−1^ after phase 1, indicating that bacterial growth was oxygen-limited (Supplementary Fig. S2 A). During days 45-72, the acetate concentration in the reactors was generally <1 mM, suggesting that it was nearly completely consumed by the bacteria.

### Nitrification in R0 was less inhibited by acute increase in carbon supply than in R1

**Figure 3:**
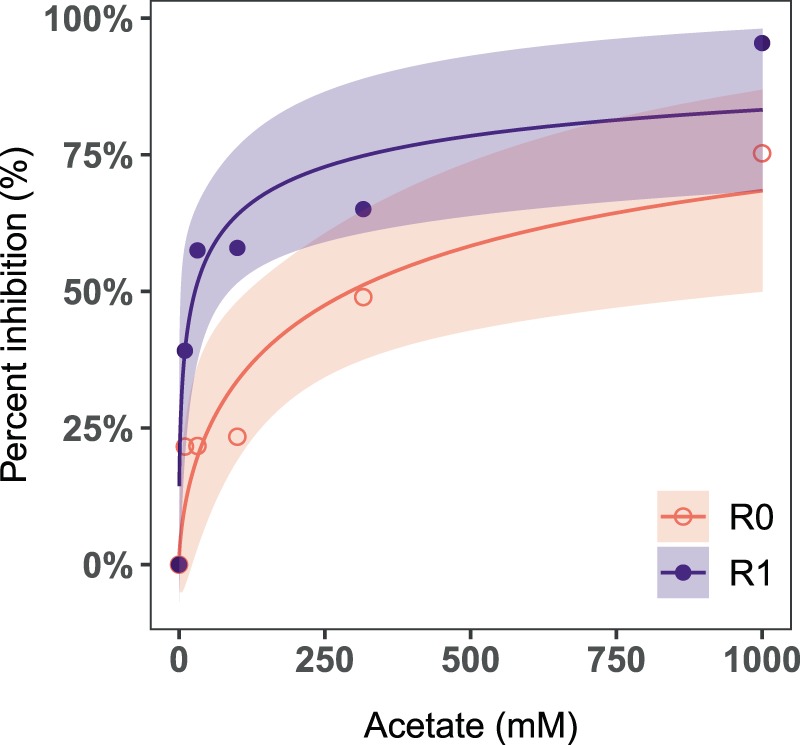
Test for inhibition of nitrification by acute carbon addition, conducted in batch reactors on day 20. The graph shows a dose-response curve for each treatment as the percent inhibition in nitrate production capacity (NPR_max_) as a function of the acetate concentration.

The short-term inhibition tests showed that the ED_50_ of NPR_max_ for R0 and R1 were 273 and 27 mM, respectively (Fig. 3). This indicates that nitrification in R0 was ten times less inhibited by the acute increase in carbon concentration than R1. Nitrite accumulated to 1–11 mgN L^−1^ during each test, suggesting that nitrite oxidation was more inhibited than ammonia oxidation. Nitrite accumulation decreased with increasing acetate concentration, likely due to lower ammonia oxidation rates at higher acetate concentrations (Supplementary Fig. S3). The nitrogen mass balance showed that less than 6% of nitrogen was unaccounted for, suggesting that nitrogen loss due to processes such as denitrification and heterotrophic uptake was negligible.

### Biofilm microbial community composition

The sequencing effort resulted in an average of 58 832 reads per sample with 1023 OTUs, thus covering 87% of the estimated richness. Despite the use of primers for bacteria, one OTU was classified as an ammonia oxidizing archaea (genus: *Nitrososphaera*). We inspected the OTU table for potential nitrifying bacteria (see Section 2.6.2 for details), and identified seven OTUs belonging to the AOB guild, five of which were classified as *Nitrosomonadaceae*. The other two OTUs were classified as likely *Nitrosomonadales* and were present at <0.2% abundance. Three OTUs were found for the NOB guild, of which two were classified as the genus *Nitrospira* and one as *Bradyrhizobiaceae*. The OTU classified as *Bradyrhizobiaceae* had a representative sequence identical to that of the type strain for *Nitrobacter vulgaris*. We therefore considered this OTU (OTU_21) to represent *Nitrobacter*.

#### The relative abundance of nitrifying taxa differed between treatments

**Figure 4:**
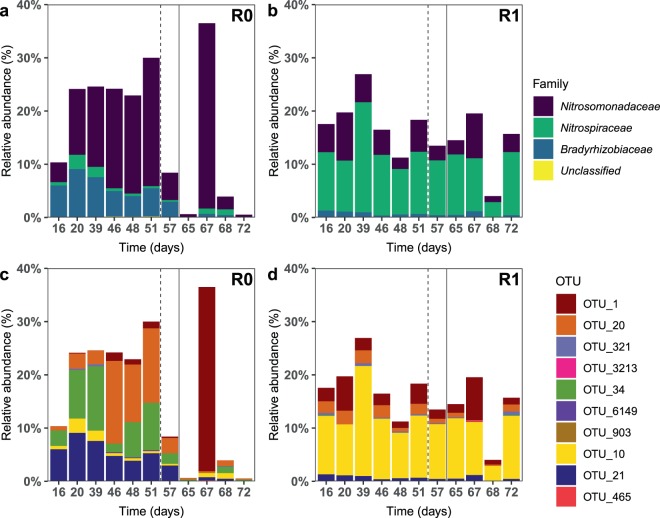
Relative abundance of nitrifying bacteria in the biofilm during the experimental period. Graphs **a** and **b** show the relative abundance of nitrifying families in treatments R0 and R1, respectively. Graphs **c** and **d** show the relative abundance of different nitrifying OTUs in treatments R0 and R1, respectively. OTUs 10, 21, and 465 were classified as nitrite oxidizing bacteria, whereas the rest were ammonia oxidizing bacteria. The influent C/N to both reactors was increased to 3 on day 52 (dashed line). The medium flow rate to both reactors was doubled on day 59 (solid line).

In phase 1, the average proportion of nitrifiers in R1 was 20% lower than in R0, likely due to the higher abundance of heterotrophs in the carbon-fed reactor (Fig. 4). A chronic increase in the carbon supply impacted the nitrifying community in R0 more severely than in R1. The proportion of nitrifiers in R0 reduced from approximately 23% to less than 8% (except on day 67, likely outlier), whereas it did not change significantly in R1. The main genus in the AOB guild was *Nitrosomonas*, while *Nitrosospira* had <1% abundance in all samples. The dominant ammonia oxidizing OTUs in R0 were OTU_34 and OTU_20 (both classified as *Nitrosomonas*), constituting up to 24% of the community (Fig. 4c). Neighbour-joining analysis of the sequences indicated that OTU_34 was closely related to *N*. *europaea*, whereas OTU_20 was closer to *N. marina*, *N. aestuarii*, and *N. oligotropha* (Supplementary Fig. S4). The abundance of *Nitrosomonas* OTUs were far lower in R1, and here OTU_34 was rare throughout the experiment (Fig. 4d). The most abundant AOB OTUs in R1 were OTU_20 and OTU_1 (related to *N. ureae*, Supplementary Fig. S4). The dominant NOB OTUs in R0 and R1 as classified by the RDP Classifier belonged to the genus *Nitrobacter* (OTU_21) and the genus *Nitrospira* (OTU_10 and 465), respectively.

#### Microbial composition in the two treatments were significantly different during phase 1

**Figure 5:**
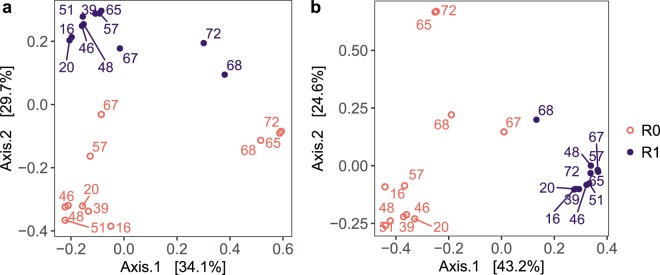
Ordination by principal coordinates analysis (PCoA) based on Bray-Curtis dissimilarity including **a**) all OTUs, and **b**) nitrifying OTUs in the biofilm samples. Labels indicate sampling day. The influent C/N to both reactors was increased to 3 on day 52. The medium flow rate to both reactors was doubled on day 59. Square brackets show percent variance explained by each coordinate axis.

Ordination by PCoA based on Bray-Curtis (relative abundance) distances showed that the microbial communities of R0 and R1 were dissimilar in phase 1 (Fig. 5a). This was also evident from the proportions of the OTUs at the order level (Supplementary Fig. S5). Further, ordination based on Sørensen-Dice (presence/absence) distances (Supplementary Fig. S6 A) showed similar trends as the ordination based on Bray-Curtis distances. This suggests that the compositional difference was due to differences in both the species inventory and in the proportions of taxa. PERMANOVA analyses confirmed that the difference in the community composition was significant (*p* = 0.002, R^2^ = 0.64). Also, the Bray-Curtis dissimilarity between the treatments was high (60–70%). SIMPER analyses showed that the OTUs contributing most to this dissimilarity belonged to the genera *Nitrospira*, *Zoogloea* (more abundant in R1), and *Nitrosomonas* (cumulative contribution ~25%, Supplementary Table S1 A).

#### Microbial composition in both treatments changed significantly after phase 1

After phase 1, the microbial community composition shifted in both reactors (Fig. 5a). The composition in phase 1 and phases 2–3 was significantly different, both in R0 (*p* = 0.002, R^2^ = 0.37) and R1 (*p* = 0.002, R^2^ = 0.26). The composition in R0 was more impacted by the increased carbon supply than in R1, as the Bray-Curtis dissimilarity between phase 1 and phase 3 was greater in R0 (83%) than in R1 (53%). The temporal shift in R0 was mainly because OTUs belonging to the genera *Acinetobacter*, *Delftia*, and *Acidovorax* increased and *Nitrosomonas* decreased (cumulative contribution ~27%, Supplementary Table S1 B), while in R1, *Delftia*, *Acidovorax*, and *Zoogloea* increased (cumulative contribution ~24%, Supplementary Table S1 C). After operation under identical (high loading) conditions for 20 days, the difference in the community composition between the reactors became insignificant (*p* = 0.1, R^2^ = 0.20) and the dissimilarity reduced from 60–70% to ~50%.

#### Nitrifying community composition in the two treatments over time

The ordination based on the nitrifying OTUs showed similar trends as for the entire community (Fig. 5b). However, the trends in the corresponding Sørensen-Dice plots were not as clear, likely due to fewer OTUs (Supplementary Fig. S6 B). Unlike the entire microbial community, the dissimilarity in the nitrifying community composition did not change significantly from phase 1 to phase 3 (~80% dissimilarity). PERMANOVA confirmed that the nitrifying communities in R0 and R1 differed significantly (*p* = 0.0001, R^2^ = 0.40) throughout the study. Moreover, in R0, the nitrifying community composition changed significantly after phase 1 (*p* = 0.002, R^2^ = 0.35), mainly due to a decrease in proportion, whereas it did not change in R1 (*p* = 0.19, R^2^ = 0.14).

#### Taxa diversity in R0 reduced after phase 1

During phase 1, R0 had marginally higher first-order diversity and significantly higher taxa richness than R1 (Supplementary Table S2). After phase 1, first-order diversity reduced significantly in R0, but not in R1. Richness reduced in both treatments. Consequently, in phases 2–3, there was no significant difference between the two treatments based on any of the diversity indices. Based on the nitrifying community, the first-order diversity was higher in R0, but the other diversity indices were not significantly different between treatments.

## Discussion

In phase 1, both reactors had similar (~100%) nitrification performance. While this is consistent with a previous study^7^, it contradicts other studies that reported a 30–70% reduction in AOX at C/N ratios of 0.6-0.9 ^16,34,35^. The discrepancy could be because in some of these studies, the same bioreactor was operated at new C/N ratios with 2-4 weeks of acclimation, whereas in the present study, two separate bioreactors were adapted to different C/N ratios for four months. Thus, over an extended period, carbon-fed nitrifying bioreactors can adapt and perform as well as autotrophic bioreactors. Despite similar activity during phase 1, the microbial community composition in the two treatments was significantly different, indicating that the C/N ratio had a deterministic effect on the microbial succession in the biofilm – including the nitrifying bacteria. The succession may have also been influenced by the biofilm thickness, as higher carbon supply leads to thicker biofilms ^8,36–38^. As the thickness influences the biofilm properties in several ways, such as oxygen diffusion and redox gradient, it can play an important role in determining the microbial community composition^14,39^. Thus, both the biofilm thickness and microbial composition likely influenced the response of the bioreactors to increases in carbon supply.

An acute increase in the dissolved organic carbon inhibited nitrification 10 times more severely in the carbon-fed reactor R1 than in R0. The nitrifying biomass in both reactors was likely equal, as the nitrification activity during phase 1 was similar. However, R1 had higher heterotrophic biomass than R0 due to the greater proportion of heterotrophs. Thus, the increase in acetate concentration stimulated heterotrophic growth to a greater extent in R1. This intensified the competition for oxygen and space, thereby inhibiting the nitrification activity more severely in R1 than in R0. To the best of our knowledge, this is the first study to model the impact of acute carbon stress on nitrifying biofilms operated at different C/N ratios. The results suggest that autotrophic conditions should be maintained in the nitrification units of water treatment systems receiving influents with varying carbon concentration, to prevent inhibition of nitrification by carbon fluctuations. Autotrophic conditions may be maintained by designing pre-treatment systems for carbon removal before the nitrification unit. Other important design variables are the loading rate per biofilm area and the oxygen supply. Apart from inducing competition, organic carbon can also directly inhibit nitrification by inactivating enzymes in the nitrification process^15,35^. But in such a scenario, we would have expected similar inhibition in both reactors, unlike in this study. The greater inhibition in R1 strongly suggests that it was due to resource competition between the nitrifiers and the heterotrophs rather than direct inhibition by organic carbon.

Under a chronic increase in carbon supply in phase 3, the AOX decreased by 14–22% (Fig. 1b). R0 had higher nitrification efficiency than R1 despite a lower proportion of nitrifiers, likely due to greater functional redundancy. The decrease in nitrification efficiency with increasing carbon supply is consistent with previous studies^7,9^, but in contrast to the 70% reduction reported by another study^34^. Further, nitrite accumulation was observed in phases 2 and 3 (and the acute inhibition tests), suggesting that despite different dominant NOB in the two reactors, the NOB were more suppressed than the AOB. This was likely because the bacterial growth was oxygen-limited. As NOB have lower specific growth rates and oxygen affinity than AOB, they would be inferior in the competition for oxygen with AOB and the prolific heterotrophs^3,40^. This observation is in contrast to a previous study where nitrite accumulation was not observed^35^, but consistent with some other studies^16,41^. In the beginning of phase 3, full carbon removal was achieved in R0 within 24 hours, indicating that autotrophic biofilms adapt quickly to consume organic carbon. This is important when designing systems where influents may have variable carbon concentration.

A sustained increase in dissolved organic carbon supply also increased denitrification activity in both reactors. On the first days of phases 2 and 3, denitrification activity was significantly greater in R1. Also, in phase 3, the long-term denitrification activity (nitrogen loss) was slightly greater in R1 (18 ± 6%) than in R0 (10 ± 6%) (excluding day 65). The values are close to a previous study that reported 18–31% nitrogen loss at low C/N ratios (0.6–2.3)^16^. The higher denitrification activity in R1 may be linked to the greater thickness of carbon-rich biofilms and more oxygen competition in R1. Under oxygen limitation, anoxic processes such as denitrification may be favoured in the presence of organic carbon^14,42^. The denitrification strategy can alleviate the competitive pressure by using nitrate instead of oxygen for respiration and by reducing the carbon available for the aerobic heterotrophs (thereby slowing growth and reducing the oxygen consumption). Therefore, under the carbon- and nitrate-rich conditions in phase 3, heterotrophs that could utilize both oxygen or nitrate for respiration had a competitive advantage, especially in the oxygen-deprived layers of the biofilm. This is also reflected in the change in the species inventory of the biofilm, as the relative abundance of potential denitrifiers *– Acinetobacter* (in R0 only), *Delftia*, and *Acidovorax*^16,43,44^ *–* increased substantially (total proportion up from <1% to 30–50%). As mentioned previously, simultaneous nitrification and denitrification can be problematic in some systems. Under low nitrate and high sulphate concentration (especially in seawater), sulphate reducing bacteria (SRB) can produce H_2_S, a health hazard in WWTP^17^ and a huge problem in marine RAS, where even low concentrations of H_2_S can kill the fish. In the past couple of years, H_2_S has been suspected as the cause of mass mortalities of fish in brackish and seawater RAS. In the present study, SRB were not detected at the assigned sequence similarity cut-off. Nonetheless, it is advisable to maintain low carbon concentration in RAS bioreactors to avoid anoxic zones in the biofilm and minimize the risk of H_2_S.

In contrast to a previous study^16^, the biofilm α-diversity (both overall and nitrifying OTUs) in the carbon-deprived reactor R0 was higher than in R1 and reduced upon increasing carbon supply. This suggests the loss of K-strategists that could not compete with r-strategists at a higher carrying capacity. By comparison, the diversity in R1 did not change during the study, even though the nitrification efficiency decreased and denitrification activity increased. The proportion of nitrifiers decreased with increasing C/N, corroborating previous studies^7,15,37^. In phase 1, the proportion of AOB in carbon-fed R1 was only 5% compared to 15% in R0, likely due to the competition for space and oxygen. Although the presence of nitrifiers throughout the biofilm have been reported^12,40,45^, the AOB *Nitrosomonas* generally prefer the oxygen-rich outer biofilm layers^14,42^. However, in the carbon-fed biofilm, heterotrophs likely dominated the outer biofilm, with the AOB underneath and the NOB in the layers below^20,36,38,46^. As the deeper strata are usually oxygen limited^40,42^, microbes with a higher oxygen affinity should have been dominant in this niche. So the prevalence of *N. oligotropha-*like taxa in R1 is surprising, as this lineage is reported to have a low oxygen affinity compared to other *Nitrosomonas* species, such as *N. europaea/eutropha*^47,48^. However, the dominant AOB in R1, *N. urea* and *N. oligotropha*, both have a higher ammonia affinity than *N. europaea/eutropha*, which may have made them superior competitors based on substrate affinity in the diffusion-limited biofilm layers^49,50^. By comparison, in the autotrophic biofilm, the dominant OTUs were similar to *N. oligotropha/aestuarii/marina* and *N. europaea*. These two taxa likely dominated different biofilm niches, as shown by another study where *N. europaea*-lineage and *N. oligotropha* coexisted on the outer biofilm layer, whereas *N. oligotropha* dominated the deeper layers^51^. Moreover, *N. europaea* was reported to support heterotrophic growth and biofilm formation in the absence of external organic carbon^52^. Thus, the greater oxygen availability in R0 selected for different taxa as opposed to oxygen-limited R1. Our results are in contrast to another study^16^, where the dominant AOB (*N. europaea*) did not change with C/N ratio, perhaps because of lesser adaptation time to the C/N ratio.

In phase 1, the dominant nitrite oxidizer in R0 belonged to the genus *Nitrobacter*. The r-strategist *Nitrobacter* likely outcompeted *Nitrospira* owing to the lesser oxygen competition (hence higher oxygen availability) in the carbon-deficient reactor^42,53,54^. *Nitrobacter* were also identified as the dominant NOB at low C/N ratios (0–2.3) in a previous study^16^. However, other studies did not detect *Nitrobacter* in autotrophic biofilms^13,20,40^, suggesting that the initial microbial community composition may influence the steady-state community^55,56^. By contrast, *Nitrospira* was the dominant nitrifier in R1, present at a relative abundance as high as 20%. *Nitrospira* preferentially reside in the interior of the biofilm, and being K-strategists, are more competitive than *Nitrobacter* under oxygen-limiting conditions^40,42,54,57^. The microaerophilic nature of *Nitrospira* may, therefore, have made it a superior competitor to *Nitrosomonas* and *Nitrobacter* in R1. Moreover, the higher proportion of NOB than AOB in R1 suggests that the *Nitrospira* may have grown by comammox or alternative growth mechanisms, which can be advantageous in ammonia-limited environments^58,59^. The ability of *Nitrospira* to co-exist with heterotrophs may also explain why it is wide-spread in natural ecosystems and is the dominant nitrite oxidizer in wastewater treatment plants^58^. Indeed, upon increasing the carbon supply, the proportion of NOB reduced greatly in R0, with a significant decrease in the proportion of *Nitrobacter* but a slight increase in the proportion of *Nitrospira*. In contrast, the increased carbon supply did not affect the NOB composition in R1.

Phase 2 was run for only seven days, which may be insufficient to confirm the long-term impact of the increased C/N. The fluctuations in ammonia, nitrite, and nitrate in the end of phase 3 suggest that steady state was not achieved, especially in R0. This indicates that the destabilizing effect of a chronic increase in carbon supply persists for at least two weeks. Also, the results in phase 3 were the combined effect of the increased carbon and nitrogen loading rate^8,12^, and decreased HRT^6^. In this study, the differences between the treatments were likely due to differences in C/N as well as the biofilm thicknesses. To unconfound biofilm thickness and C/N ratio, future studies should compare the impact of organic carbon on biofilms with controlled thickness, such as in Z-carriers^11^.

In conclusion, this study showed that autotrophic bioreactors were 10 times less inhibited by acute increase in organic carbon than carbon-fed bioreactors – likely due to differences in the heterotrophic potential. A sustained increase in carbon supply and substrate loading reduced nitrification efficiency and increased denitrification to a greater extent in carbon-fed biofilms. The chronic increase in carbon supply also increased the proportion of potential denitrifiers in both reactors. The results suggest that autotrophic conditions are preferable in nitrifying bioreactors when acute carbon fluctuations are expected and/or anoxic processes are undesirable, such as in RAS. This implies that nitrification systems should be designed to maintain high nitrifying to heterotrophic biomass ratio, for e.g., by pre-treatment to remove organic carbon before the nitrifying unit.

## Supplementary information

Supplementary Information.
