# Supplementary material for: Nitrifying biofilms deprived of organic carbon show higher functional resilience to increases in carbon supply
Source: Sci Rep. 2020 Apr 28;10:7121. doi: 10.1038/s41598-020-64027-y (PMC7189377; doi:10.1038/s41598-020-64027-y)
Supplement: Supplementary file 1 — Supplementary Information. [file 41598_2020_64027_MOESM1_ESM.pdf]

Supplementary Information for

Nitrifying biofilms deprived of organic carbon show higher functional resilience to increases in carbon supply

Sharada Navada[1,2]*, Maja F. Knutsen[3,a], Ingrid Bakke[3], Olav Vadstein[3]

[1] Department of Chemistry, NTNU - Norwegian University of Science and Technology, N-7491 Trondheim, Norway

[2] Krüger Kaldnes AS (Veolia Water Technologies), N-3241 Sandefjord, Norway

[3] Department of Biotechnology and Food Science, NTNU - Norwegian University of Science and Technology, N-7491 Trondheim, Norway

[a] Present address: Oxy Solutions, Gaustadalleen 21, N-0349 Oslo, Norway

*Corresponding author: sharada.navada@ntnu.no (Sharada Navada)

Figure S1: Ammonia oxidation capacity tests conducted in phase 1 showing the ammonia concentration as a function of time. Maximum ammonia oxidation rate (AOR_{max}) was calculated from the slope of the regression lines. The tests were conducted on biofilm carriers from R0 and R1 in synthetic media with C/N ratios of 0 and 1 in batch reactors.

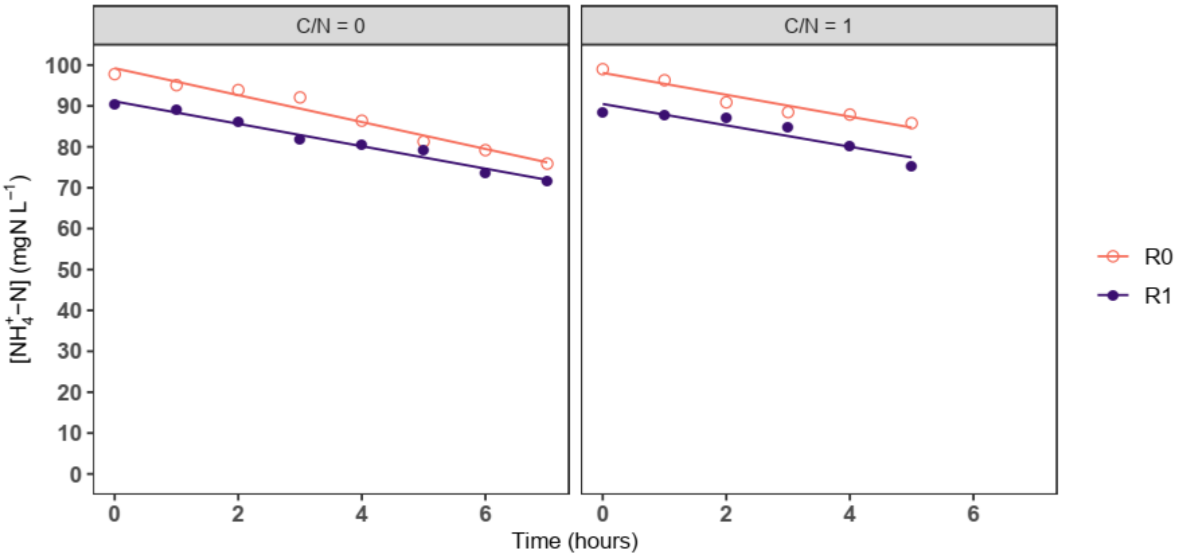

Figure S2: Dissolved oxygen concentration in reactors R0 and R1 during A) during days 40-72, B) the first nine hours of phase 2, and C) the first eight hours of phase 3. In phase 1 (days 1-52), R0 and R1 were operated at C/N ratios of 0 and 1, respectively. Phase 2 was started on day 52 by increasing the influent C/N ratio to 3 in both reactors (dashed line). Phase 3 was started on day 59 by doubling the synthetic medium flow rate to both reactors (solid grey line).

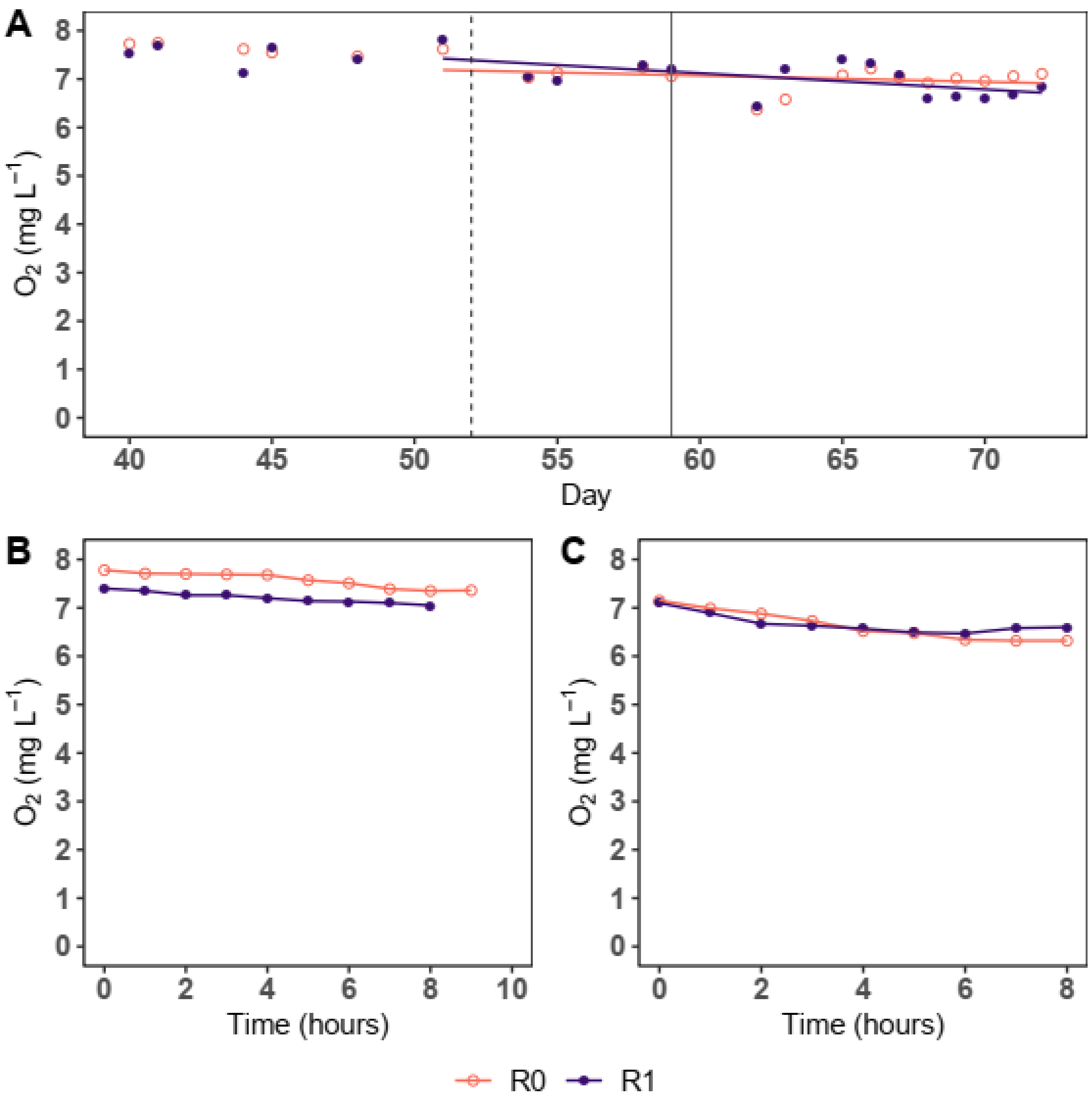

Figure S3: Nitrite accumulation during the acute carbon inhibition tests on day 20. The graph shows the difference between the initial and final nitrite concentration during the capacity tests at different acetate concentrations. The initial nitrite concentration was less than 0.5 mgN L^{-1} at all acetate concentrations.

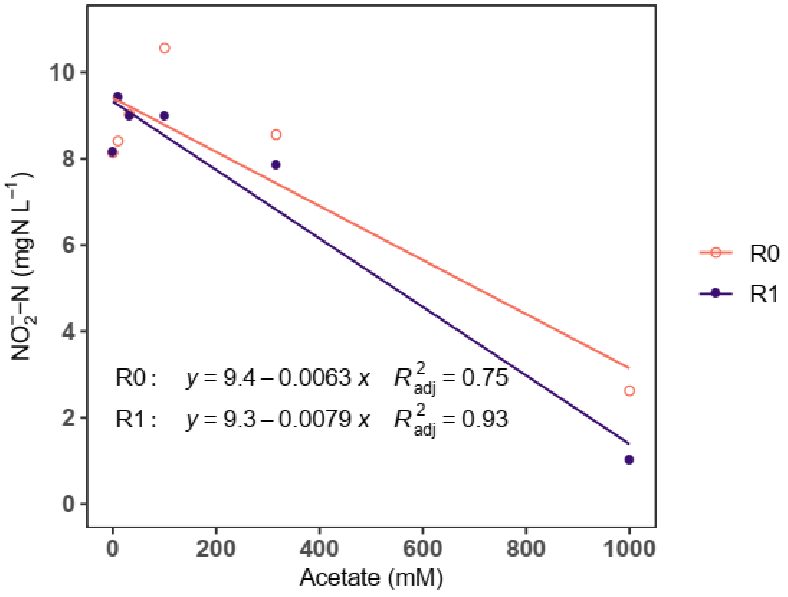

Figure S4: A neighbor-joining showing the relationships between the *Nitrosomonadaceae* OTUs identified in this study and *Nitrosomonas* Type strains. Type strain sequences were downloaded from the Ribosomal Database Project.

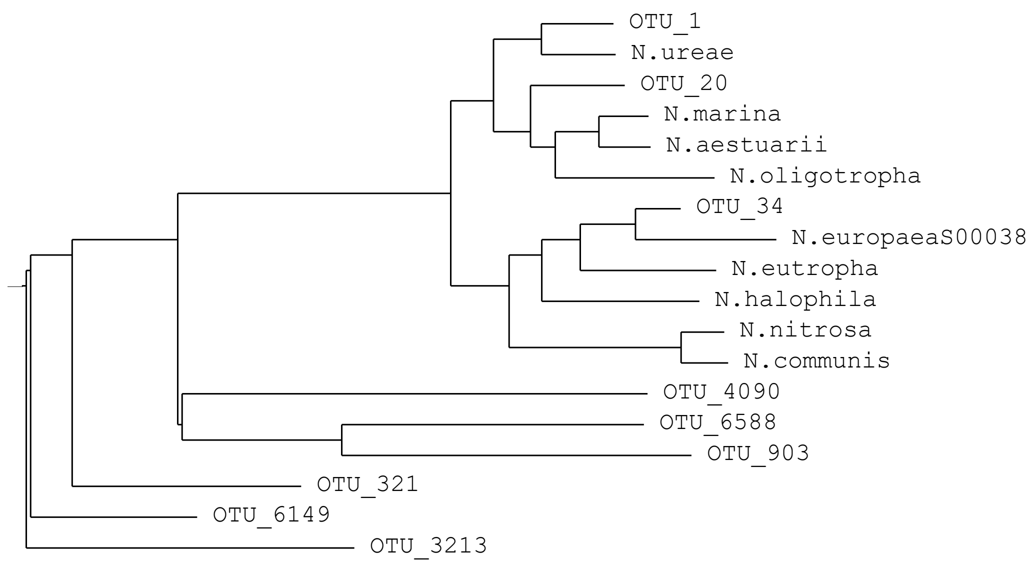

Figure S5: Relative abundance of bacteria on the biofilm carriers in A) R0 and B) R1. The results are shown at the taxonomic level of order. The dotted and solid vertical lines indicate the transition to phase 2 (day 52) and phase 3 (day 59), respectively.

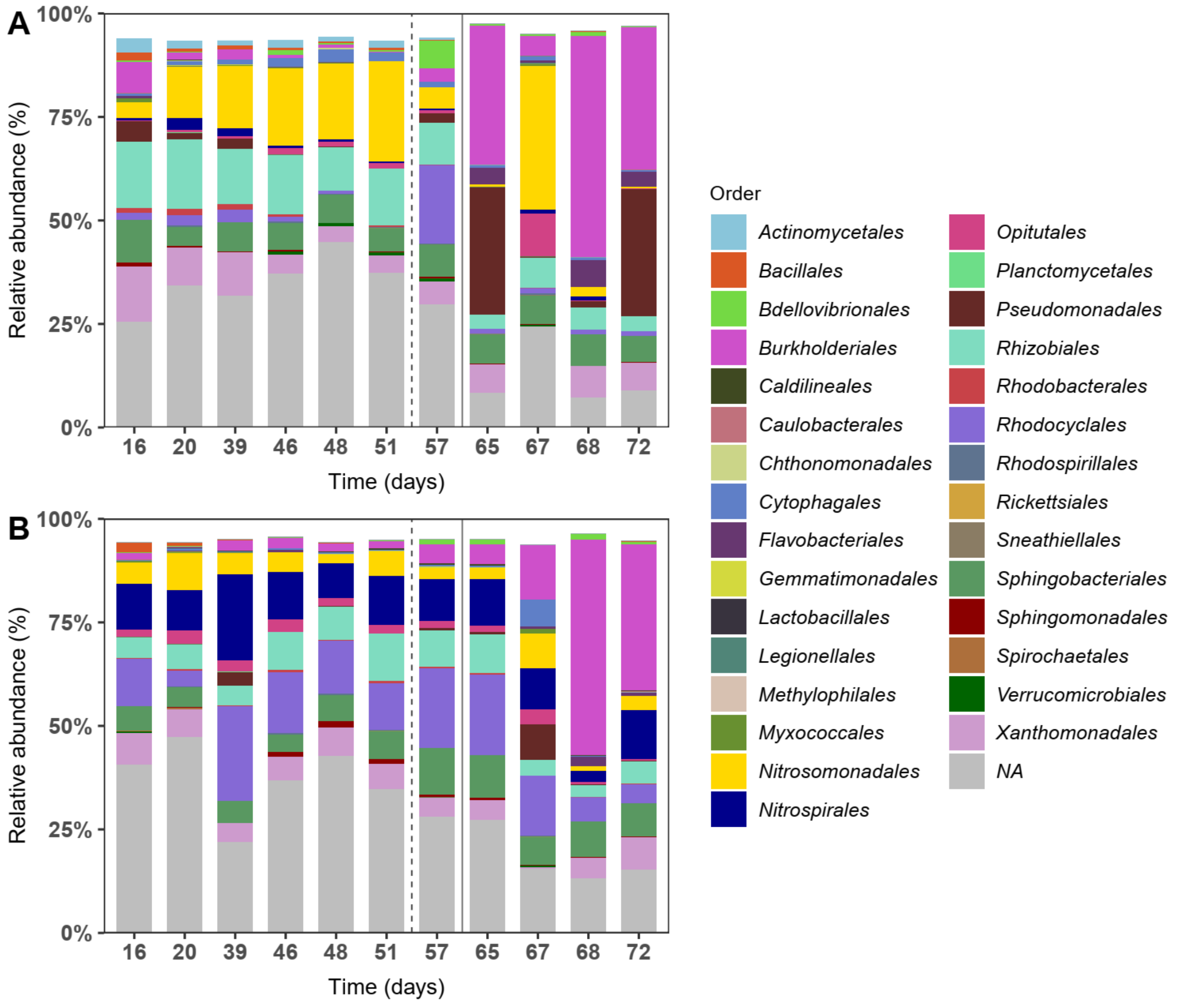

Figure S6: Ordination by PCoA based on Sørensen-Dice indices (presence/absence) including A) all OTUs, and B) nitrifying OTUs. Labels indicate sampling day. The influent C/N to both reactors was increased to 3 on day 52. The synthetic medium flow rate to both reactors was doubled on day 59. Square brackets show percent variance explained by each coordinate axis.

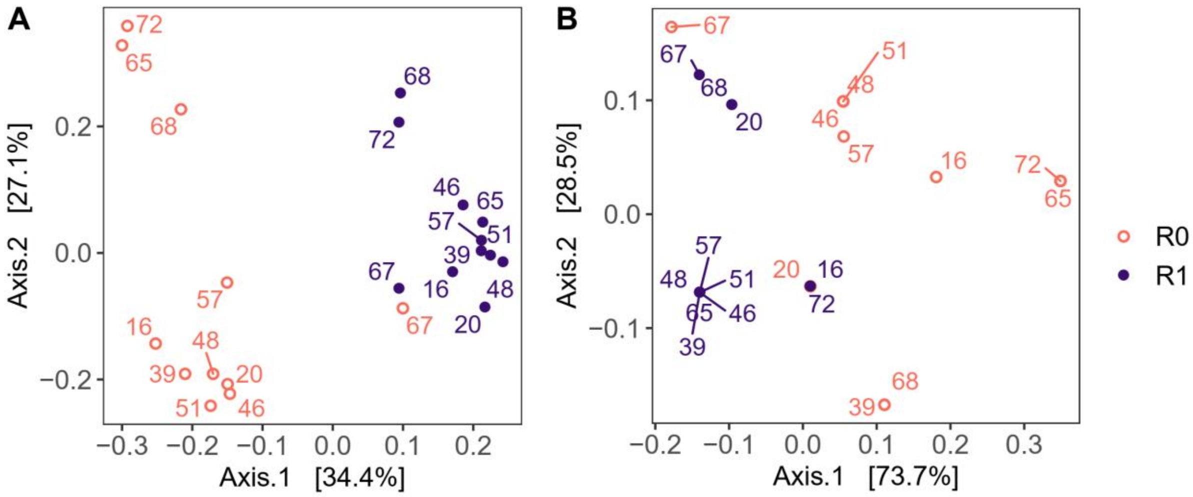

Table S1: SIMPER analyses showing the OTUs with highest contribution to dissimilarity between microbial community composition in the following situations. Average relative abundance of the OTUs in each treatment or phase is shown, along with its contribution to the dissimilarity.

A) R0 and R1 in phase 1

		Average relative abundance			
**OTU**	**Genus**	**R0**	**R1**	**Contribution (%)**	**Cumulative contribution (%)**
**OTU_10**	*Nitrospira*	1%	12%	9%	9%
**OTU_5**	*Zoogloea*	0%	8%	6%	15%
**OTU_34**	*Nitrosomonas*	7%	0%	5%	20%
**OTU_3**[1]	*NA*	12%	7%	5%	26%
**OTU_20**	*Nitrosomonas*	8%	2%	5%	30%

B) phase 1 and after phase 1 in treatment R0

		Average relative abundance			
**OTU**	**Genus**	**Phase 1**	**Phases 2-3**	**Contribution (%)**	**Cumulative contribution (%)**
**OTU_12**	*Acinetobacter*	0%	13%	9%	9%
**OTU_2**	*Delftia*	0%	11%	8%	16%
**OTU_6**	*Acidovorax*	0%	9%	6%	22%
**OTU_3**	NA	12%	5%	6%	28%
**OTU_1**[3]	*Nitrosomonas*	1%	7%	5%	33%
**OTU_20**	*Nitrosomonas*	8%	1%	5%	38%

C) phase 1 and after phase 1 in treatment R1

		Average relative abundance			
**OTU**	**Genus**	**Phase 1**	**Phases 2-3**	**Contribution (%)**	**Cumulative contribution (%)**
**OTU_2**	*Delftia*	1%	11%	12%	12%
**OTU_9**[2]	NA	10%	4%	6%	18%
**OTU_6**	*Acidovorax*	1%	6%	6%	25%
**OTU_11**	*Zoogloea*	4%	7%	6%	30%
**OTU_3**	NA	7%	3%	5%	35%

[1,2] OTU_3 could not be classified beyond the domain level. OTU_9 was classified as belonging to phylum *Ignavibacteriae*.

[3] OTU_1 had unexpectedly high abundance in R0 on day 67, likely an outlier

Table S2: Diversity of the biofilm microbial community (based on all OTUs and OTUs representing nitrifiers) in R0 and R1 during and after phase 1. The α-diversity was determined as the first-order diversity (N1), richness (N0, zero-order diversity,), and evenness (N1/N0). Significant differences ($p < 0.05$) between the treatments are marked in bold with an asterisk (*).

	In phase 1			***After phase 1***		
ALL OTUs	**R0**	**R1**	***p***	**R0**	**R1**	***p***
First-order diversity (N_1)	33 ± 8	26 ± 3	0.06	18 ± 7	23 ± 5	0.22
Richness (N_0)	83 ± 7	70 ± 2	**0.001***	59 ± 10	63 ± 7	0.42
Evenness (N_1/N_0)	0.4 ± 0.1	0.4 ± 0.04	0.46	0.3 ± 0.1	0.4 ± 0.1	0.16
NITRIFIERS	**R0**	**R1**	***p***	**R0**	**R1**	***p***
First-order diversity (N_1)	3.3 ± 0.3	2.4 ± 0.2	**0.02***	3.0 ± 1.1	2.5 ± 0.1	0.34
Richness (N_0)	5.5 ± 0.8	5.0 ± 0.6	0.27	4.4 ± 1.3	5.0 ± 0.7	1.00
Evenness (N_1/N_0)	0.6 ± 0.1	0.5 ± 0.05	0.35	0.7 ± 0.3	0.5 ± 0.1	0.30
